# A Protein-Based Pentavalent Inhibitor of the Cholera Toxin B-Subunit**

**DOI:** 10.1002/anie.201404397

**Published:** 2014-07-02

**Authors:** Thomas R Branson, Tom E McAllister, Jaime Garcia-Hartjes, Martin A Fascione, James F Ross, Stuart L Warriner, Tom Wennekes, Han Zuilhof, W Bruce Turnbull

**Affiliations:** School of Chemistry and Astbury Centre for Structural Molecular Biology, University of Leeds, LeedsLS2 9JT (UK); Laboratory of Organic Chemistry, Wageningen UniversityDreijenplein 8, 6703 HB Wageningen (The Netherlands); Department of Chemical and Materials Engineering, King Abdulaziz UniversityJeddah (Saudi-Arabia)

**Keywords:** carbohydrates, glycoproteins, multivalency, protein modifications, protein structures

## Abstract

Protein toxins produced by bacteria are the cause of many life-threatening diarrheal diseases. Many of these toxins, including cholera toxin (CT), enter the cell by first binding to glycolipids in the cell membrane. Inhibiting these multivalent protein/carbohydrate interactions would prevent the toxin from entering cells and causing diarrhea. Here we demonstrate that the site-specific modification of a protein scaffold, which is perfectly matched in both size and valency to the target toxin, provides a convenient route to an effective multivalent inhibitor. The resulting pentavalent neoglycoprotein displays an inhibition potency (IC_50_) of 104 pm for the CT B-subunit (CTB), which is the most potent pentavalent inhibitor for this target reported thus far. Complexation of the inhibitor and CTB resulted in a protein heterodimer. This inhibition strategy can potentially be applied to many multivalent receptors and also opens up new possibilities for protein assembly strategies.

Many viruses, bacteria, and protein toxins adhere to their target cells by binding to specific cell-surface carbohydrates. Diarrheal diseases that are initiated in this way account for around two million deaths annually.[1] For example, cholera is caused by an AB_5_ protein toxin that has a single toxic A-subunit associated with five nontoxic B-subunits (CTB), which constitute a pentameric receptor for the GM1 glycolipid found on the surface of intestinal epithelial cells.[2] Multivalent binding between CTB and up to five copies of its GM1 ligand facilitates the entry of the toxin into the cell by endocytosis.

Multivalency is a common feature of cell-surface adhesion and has thus provided the primary focus for creating inhibitors of carbohydrate-binding proteins, including CTB.[3] Polymeric structures and dendrimers with either galactosyl residues or the more strongly binding GM1 oligosaccharide (GM1os) ligands have demonstrated the potential improvements in activity that multivalency can provide.[4] Pentavalent star-shaped structures that match the valency and positioning of the ligand groups to CTB have been shown to be some of the most successful inhibitors. A pentacyclen core with galactose ligands was used by Fan and co-workers to produce an inhibitor with a reported IC_50_ value of 1.4 μm.[5] Their modular approach showed that matching precisely the size and spacing of the ligands to the binding sites of CTB could optimize the inhibitory potential.[5b, 6] Other recent studies have used GM1os ligands on both corannulene[7] and calixarene cores.[8] These pentavalent structures gave IC_50_ values down to 5 nm and 450 pm, respectively. A pentameric carbohydrate core has also been used in the highly effective Starfish ligands for shiga-like toxin.[9] While synthetic pentavalent ligands have proven to be effective inhibitors, the synthesis of large (>5 nm diameter), precisely defined multivalent scaffolds is very challenging and hampers larger-scale applications.

Here we report a multivalent inhibitor for cholera toxin, based on an inactive mutant CTB protein that is modified with GM1os ligands (Figure 1). As the majority of the structure comes as a ready-made building block, the synthetic route to the inhibitor is relatively short. Previous attempts to introduce sugars at defined sites on an ankyrin repeat protein[10] and the barstar protein[11] led to ligands and inhibitors of plant lectins. However, as no attempts were made to match the size and spacing of the ligands to the binding sites, these multivalent compounds led to only modest enhancements in activity and/or lectin aggregation, which may be undesirable. In the example reported here, the pentavalent inhibitor is matched to the size and valency of the target CTB protein, which leads to defined complexes and potent inhibition.

**Figure 1:**
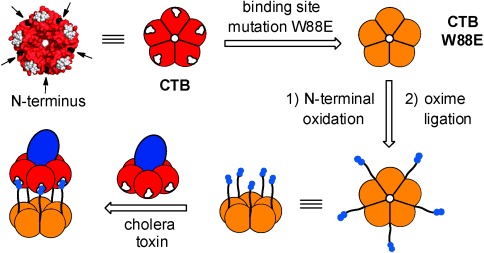
Strategy for re-engineering the CTB protein to prepare a pentavalent neoglycoprotein inhibitor for cholera toxin. The N termini of a nonbinding CTB mutant W88E are oxidized to give aldehydes that undergo oxime ligation with a carbohydrate ligand that bears an aminooxy function. The resulting neoglycoprotein has ligand groups arranged with optimal spacing to bind to the cholera toxin protein. In the 3D structure of CTB (PDB code: 3CHB),[24] the binding sites are marked in white, and N-terminal threonine residues are marked with black arrows.

We postulated that by using an inactive mutant CTB protein as the scaffold, once modified with carbohydrates, the resulting pentavalent ligand should provide a precise fit of the ligand groups with the spacing and configuration of binding sites on wild-type CTB (Figure 1). The CTB homopentamer has five N-terminal threonine residues situated on the protein surface toward the ligand-binding face and equally spaced around the protein between the GM1 binding sites. These residues bear unique vicinal amino alcohol groups, which can be selectively converted by oxidation to an aldehyde before reaction with an oxyamine.[12] Modification of the protein scaffold at these sites with five GM1os ligands would result in a pentavalent ligand. As proteins of this class can be prepared in large quantities, the synthesis is potentially scalable.[13]

The GM1os ligand was prepared by first synthesizing Boc-protected aminooxy alkyne **3** (Scheme 1) according to a reported procedure.[14] GM1 azide **2** was prepared using a chemo-enzymatic approach[15] before ligation to aminooxy alkyne **3** employing a copper-catalyzed alkyne azide cycloaddition (CuAAC) at room temperature with stirring for 48 hours. Attempts were made to use microwave-assisted CuAAC for the synthesis,[16] but proved unsuccessful in this case. After purification by reverse-phase column chromatography, the GM1os ligand **4** was isolated. The Boc group was removed with TFA to form oxyamine **5**, directly prior to the attachment to the protein without further purification (to preserve the highly reactive aminooxy group).

**Scheme 1:**
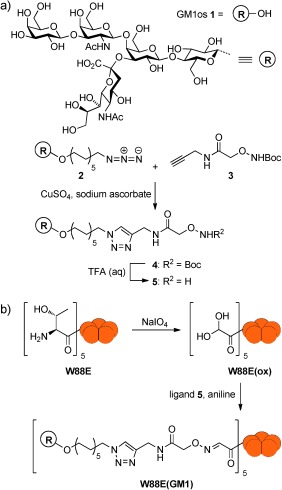
a) Synthesis of an oxime-modified GM1 oligosaccharide ligand 5; b) N-terminal oxidation of the CTB W88E protein and ligation with ligand 5.

To create the protein scaffold, a nonbinding mutant of CTB was required. Based on the work of Jobling and Holmes,[17] a tryptophan residue (W88) in the GM1 binding pocket was changed to a glutamic acid residue by site-directed mutagenesis. The resulting CTB mutant protein (**W88E**) was no longer capable of binding to GM1os (Figure S3 in Supporting Information). The five N-terminal vicinal amino alcohol groups in **W88E** were then oxidized with NaIO_4_ (Scheme 1) to give aldehydes, which were observed in their hydrated form **W88E(ox**) by ESI–MS (found: 11 557.8 Da; calcd: 11 557.9 Da). The oxidized protein was then allowed to react with deprotected oxyamine **5** in the presence of aniline at pH 7. Aniline is known to be an effective catalyst of oxime formation at pH 4.5,[18] however, as the CTB protein denatures under acidic conditions, a neutral pH value was used and the reaction still proceeded to completion within 24 hours. Pentavalent ligand **W88E(GM1)** was purified by size-exclusion chromatography (SEC) and ESI–MS confirmed that the modified protein had a mass of 12 844.5 Da (calcd: 12 844.4 Da; Figure S1). SDS-PAGE confirmed that the modification did not disrupt the pentameric form of the protein and no protein aggregation was observed (Figure S1).

An enzyme-linked lectin assay (ELLA) was performed to determine the inhibitory potential of the neoglycoprotein.[7,8] The ability of CTB to bind to a GM1-coated microtiter plate was assessed across a wide range of inhibitor concentrations. Pentavalent ligand **W88E(GM1)** exhibited an exceptionally low IC_50_ value of 104 pm (Figure 2 and Table 1), making it the most potent pentavalent ligand reported thus far. This IC_50_ value corresponds to a 5100-fold enhancement compared to monovalent GM1os **1**, or 14 300-fold enhancement compared to monovalent GM1 azide **2**. The unmodified **W88E** protein as a control showed no inhibition of CTB binding to a GM1-coated microtiter plate.

**Figure 2:**
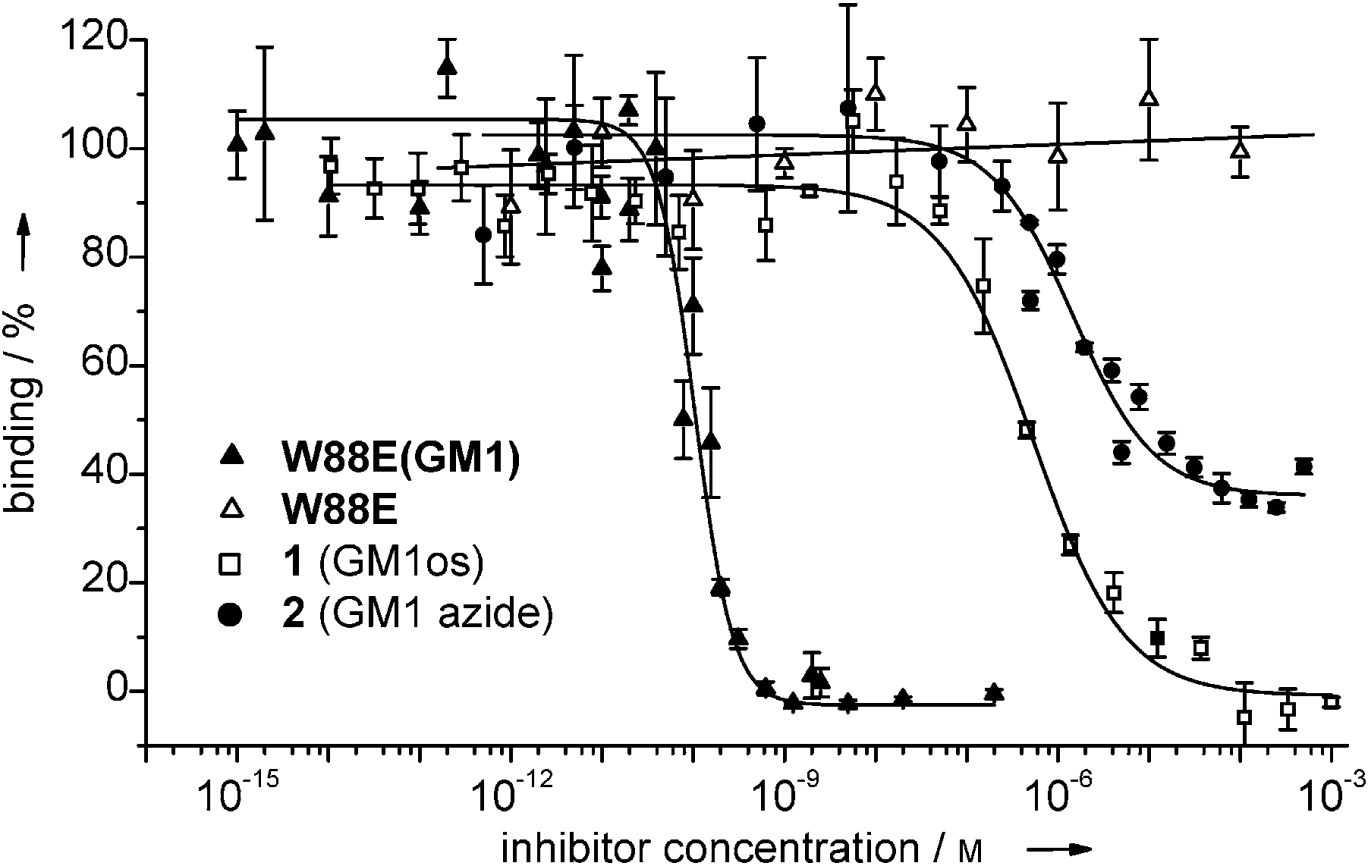
Enzyme-linked lectin assay (ELLA) indicates the inhibitory potential of W88E(GM1) and analogous monovalent ligands 1 and 2. Error bars indicate the standard error of three measurements.

**Table 1:** Inhibitory potential results from the enzyme-linked lectin assay (ELLA).

Inhibitor	Valency	log(IC_50_)	IC_50_ [nm][a]	Relative potency (per GM1 group)[b]	Hill coefficient
**2** (GM1 azide)	1	−5.83±0.16	1460	1 (1)	1.0±0.2
**1** (GM1os)	1	−6.27±0.04	530	2.75 (2.75)	0.9±0.1
**W88E(GM1)**	5	−9.98±0.08	0.104	14 300 (2860)	2.1±0.5
**W88E**	0	–[c]	–[c]	–[c]	–[c]

[a] As curve fitting was performed with log[inhibitor] as x values, the fitting errors for IC_50_ values became asymmetric about the mean and were omitted for simplicity.

[b] Potency was measured relative to monovalent GM1 azide **2**.

[c] No inhibition detected.

Isothermal titration calorimetry (ITC) was used to analyze the interaction between pentavalent protein-based inhibitor **W88E(GM1)** and wild-type CTB. **W88E(GM1)** was titrated into a solution of CTB and the expected binding stoichiometry of one GM1os ligand per binding site was observed, which is consistent with a binding model in which **W88E(GM1)** forms a protein heterodimer complex with CTB. However, an apparent *K*_d_ of 30 nm was detected, similar to that of the monovalent GM1 azide **2**, which had a *K*_d_ of 56 nm (Figure S3 and Table S1), and that of GM1os **1** (43 nm), which was used in previous studies.[20] The similarity between the affinities of mono- and pentavalent ligands contrasts with affinity enhancements reported for other multivalent systems.[21] However, the result is in line with our previous studies of GM1-based dendrimers,[22] in which it was concluded that a mismatch in valency between dimeric or tetrameric inhibitors and a pentavalent protein resulted in an aggregative mechanism of inhibition. In contrast, Fan and co-workers reported that their pentavalent ligands gave only 1:1 complexes in dynamic light scattering (DLS) and crystallization studies, and no larger aggregates were observed.[23] Therefore, DLS and analytical ultracentrifugation (AUC) experiments were used to determine if 1:1 complexes or larger aggregates had formed between CTB and **W88E(GM1)**.

DLS was first used to determine the size of the assemblies (Figure 3 a). The crystal structure of CTB shows the pentamer to have a diameter of 6.5 nm and a depth of 3.5 nm.[24] These measurements are consistent with the experimental hydrodynamic diameter of 5.6 nm. At a 1:1 ratio of CTB and **W88E(GM1)**, DLS showed a single peak at 8.4 nm. A face-to-face dimer would have dimensions of 6.5 nm by at least 7 nm, which was in agreement with the result from DLS. Increasing the ratio of CTB to **W88E(GM1)** to 5:1 gave a similar result with no larger aggregates. The observations from the DLS experiment indicated that only a protein heterodimer of pentamers is formed.

**Figure 3:**
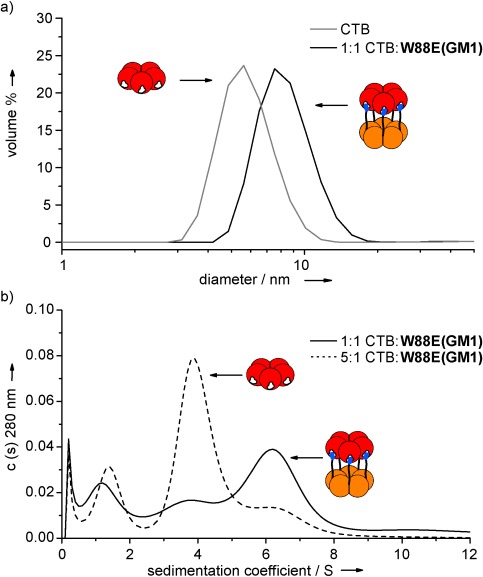
a) Dynamic light scattering (DLS) results showing the increase in the size of the particles when CTB is mixed at a ratio of 1:1 with W88E(4). b) Sedimentation coefficient distributions [c(s)] of CTB mixed with W88E(4) at different ratios and the appearance of a single species corresponding to a protein heterodimer.

Sedimentation velocity AUC was used to further study the oligomerization state of the protein complexes being formed by ligand binding. At a ratio of 1:1 CTB and **W88E(GM1)**, AUC showed two peaks with sedimentation coefficients of 6.3 S and 3.6 S, corresponding to masses of approximately 110 kDa and 50 kDa, respectively (Figure 3 b), which are consistent with the predicted masses of a dimer of protein pentamers and the single pentamer. The smaller peak at 3.6 S was likely due to the concentrations of the two components not being exactly equal. When the concentration of ligand **W88E(GM1)** was decreased, so that CTB was now in excess at a ratio of 5:1, the peak for the protein pentamer dominated, but the peak for a dimer of pentamers was still seen. This result showed that even with an excess of CTB, the only protein complex observed was a heterodimer of protein pentamers. No larger peaks were observed in the AUC experiment and there was negligible change in the protein absorbance after the AUC experiments, indicating that no protein was lost through the formation and pelleting of large aggregates.[22] Nevertheless, it is still possible that at the inhibitor concentration used in these biophysical experiments (ca. 50 000 times higher than the IC_50_ concentration for **W88E(GM1)** in the ELLA), random aggregates could initially form with modest binding affinities, as observed in our ITC experiments.[25] If those aggregates were to rearrange more slowly to form the thermodynamically preferred 1:1 complexes observed by DLS and AUC,[26] then this latter process would probably be undetectable during the ITC experiment, as the net enthalpy change for reorganization of the aggregates would probably be very small. It would be expected that 1:1 complexes should form directly at the much lower concentrations of **W88E(GM1)** inhibitor used in the ELLA.

SDS-PAGE performed on the AUC samples showed bands for the pentameric protein and a dimer of pentamers in the same ratios observed in the AUC experiments for the 1:1 and 5:1 mixtures of CTB and **W88E(GM1)** (Figure S1). Therefore, the interactions forming the protein heterodimers were sufficiently strong to survive the denaturing conditions of SDS-PAGE.

In conclusion, the combination of DLS, AUC, and SDS-PAGE confirmed that protein-based pentavalent ligand **W88E(GM1)** bound to CTB in a 1:1 ratio, forming protein heterodimers. With an IC_50_ value of 104 pm, this inhibitor is the most potent pentavalent structure described thus far. By using a protein scaffold that matches the size and the spacing between binding sites and ligands, it is possible to control the structure of the complexes that are formed.[3c] With the growing interest in ligand-mediated protein assembly, it is possible that tailored glycoproteins of this type could also be used for the preparation of nanostructured protein materials.[27] Furthermore, the protein-based inhibitor reported here also has the advantage that it is relatively easy to synthesize. Proteins of this type can be produced on an industrial scale[13] and the reaction to modify the protein is simple. While the synthesis of the carbohydrate moiety itself is not trivial,[15] the combined use of protein modification and enzymatic oligosaccharide synthesis provides an attractive strategy for biopharmaceutical synthesis. This work demonstrates a general strategy for the creation of multivalent inhibitors of protein/carbohydrate interactions.
