# Supplementary material for: A Protein-Based Pentavalent Inhibitor of the Cholera Toxin B-Subunit
Source: Angew Chem Int Ed Engl. 2014 Jul 2;53(32):8323–7. doi: 10.1002/anie.201404397 (PMC4499251; doi:10.1002/anie.201404397)
Supplement: Supplementary file 1 [file anie0053-8323-sd1.pdf]

Angewandte Chemie
Eine Zeitschrift der Gesellschaft Deutscher Chemiker

Supporting Information
© Wiley-VCH 2014
69451 Weinheim, Germany

A Protein-Based Pentavalent Inhibitor of the Cholera Toxin B-Subunit**

*Thomas R. Branson, Tom E. McAllister, Jaime Garcia-Hartjes, Martin A. Fascione, James F. Ross, Stuart L. Warriner, Tom Wennekes, Han Zuilhof, and W. Bruce Turnbull**

anie_201404397_sm_miscellaneous_information.pdf

Supporting Information

Contents

- Synthesis of 4 page 2
- DNA mutations, protein overexpression, purification and sequences page 5
- General protein modification procedures page 6
- ELLA page 8
- ITC page 10
- DLS page 12
- AUC page 12

Synthesis compound 4

4-{*N*-[2-(*tert*-butoxycarbonylaminooxy)acetamidyl]-methyl}-1-{11-[β-D-galactopyranosyl-(1→3)-2-acetamido-2-deoxy-β-D-galactopyranosyl-(1→4)-[5-acetamido-3,5-dideoxy-α-D-*glycero*-D-*galacto*-nonulopyranulosonyl-(2→3)]-β-D-galactopyranosyl-(1→4)-β-D-glucopyranosyloxy]undecyl}-[1,2,3]triazole

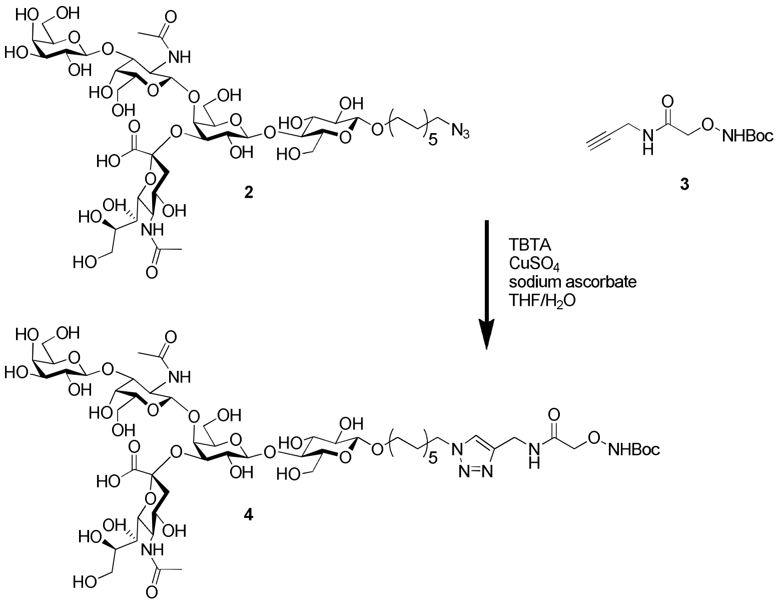

11-Azidoundecyl β-D-galactopyranosyl-(1→3)-2-acetamido-2-deoxy-β-D-galactopyranosyl-(1→4)-[5-acetamido-3,5-dideoxy-α-D-*glycero*-D-*galacto*-nonulopyranulosonyl-(2→3)]-β-D-galactopyranosyl-(1→4)-β-D-glucopyranoside **2**[1] (5.2 mg, 4.4 μmol) was dissolved in H_2O (100 μL) in a 1.5 mL Eppendorf tube. A solution of tris-(benzyltriazolylmethyl)amine (TBTA) in THF (10 mg/mL, 70 μL, 0.7 mg, 1 μmol) was added to 2-[*N*-(*t*-butoxycarbonyl)aminooxy]-*N*-(prop-2-ynyl)acetamide **3**[2] (2.0 mg, 9 μmol) and the resultant solution added to the Eppendorf. The vessels were rinsed with THF (2 × 115 μL) and the washes also added to the Eppendorf. $CuSO_4$ (1 mg/mL in H_2O, 100 μL, 0.1 mg, 0.6 μmol) and sodium ascorbate (10 mg/mL in H_2O, 40 μL, 0.4 mg, 2 μmol) were added, followed by H_2O (60 μL), and the mixture was stirred at rt for 16 h. At this point, analysis of the reaction mixture by TLC and LCMS showed no product formation so further portions of $CuSO_4$ (10 mg/mL in H_2O, 5 μL, 0.5 mg, 3.1 μmol) and sodium ascorbate (100 mg/mL in H_2O, 10 μL, 1 mg, 5 μmol) were added and stirring continued. After a further 16 h, analysis by LCMS suggested complete conversion to the expected product. The reaction was diluted with H_2O to a total volume of 12 mL and passed down a sep-pak column (pre-washed with dioxane and then equilibrated with H_2O). The column was then eluted with a step-wise gradient of aqueous dioxane (0-100% in 10% steps, 1.1 mL per wash). The column flow-through and 0-40% fractions were identified as containing the product by LCMS and TLC though the 30% and 40% fractions

also contained TBTA. The flow-through, 0, 10 and 20% fractions were combined and lyophilised to leave triazole **4** as a colourless amorphous solid (3.15 mg, 2.2 μmol, 50%)

NMR: δ_H (500 MHz, D_2O); selected assignments: 7.9 (brm, 1H, triazole H-5), 4.46-4.49 (m, 2H, Gal-B H-1, Gal-D H-1), 4.41 (d, 1H, *J* 8.0 Hz, Glc-A H-1), 4.33-4.35 (brs, 2H, CH_2-ON), 3.4-4.1 (m, 33H), 3.30 (t, 1H, J 8.7 Hz), 3.22 (t, 1H, J 8.2 Hz), 2.60 (dd, 1H, *J* 12.5 Hz, 3.5 Hz, αNeu5Ac H-3eq), 1.97 (s, 3H, Neu5Ac CH_3), 2.02 (s, 3H, GalNAc-C CH_3), 1.87 (t, 1H, *J* 12.5 Hz, αNeu5Ac H-3ax), 1.81 (m, 2H, CH_2), 1.55 (m, 2H, CH_2), 1.34 (s, 9H, Boc), 1.15-1.25 (m, 18H, CH_2); **HRMS**: found $[M+Na]^+$ 1444.6143, $C_{58}H_{99}N_7NaO_{33}$ requires 1444.6176.

HPLC:

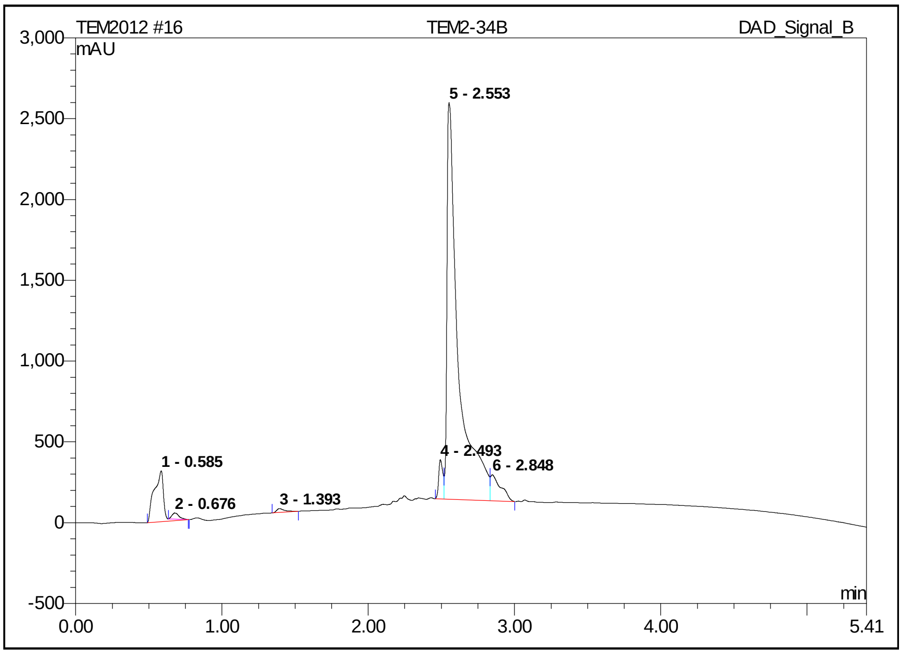

No.	Ret.Time min	Peak Name	Height mAU	Area mAU*min	Rel.Area %
1	0.58	n.a.	314.805	23.766	8.88
2	0.68	n.a.	38.331	2.145	0.80
3	1.39	n.a.	23.906	1.546	0.58
4	2.49	n.a.	241.931	8.012	2.99
5	2.55	n.a.	2454.481	219.092	81.83
6	2.85	n.a.	161.202	13.188	4.93
**Total:**			3234.656	267.749	100.00

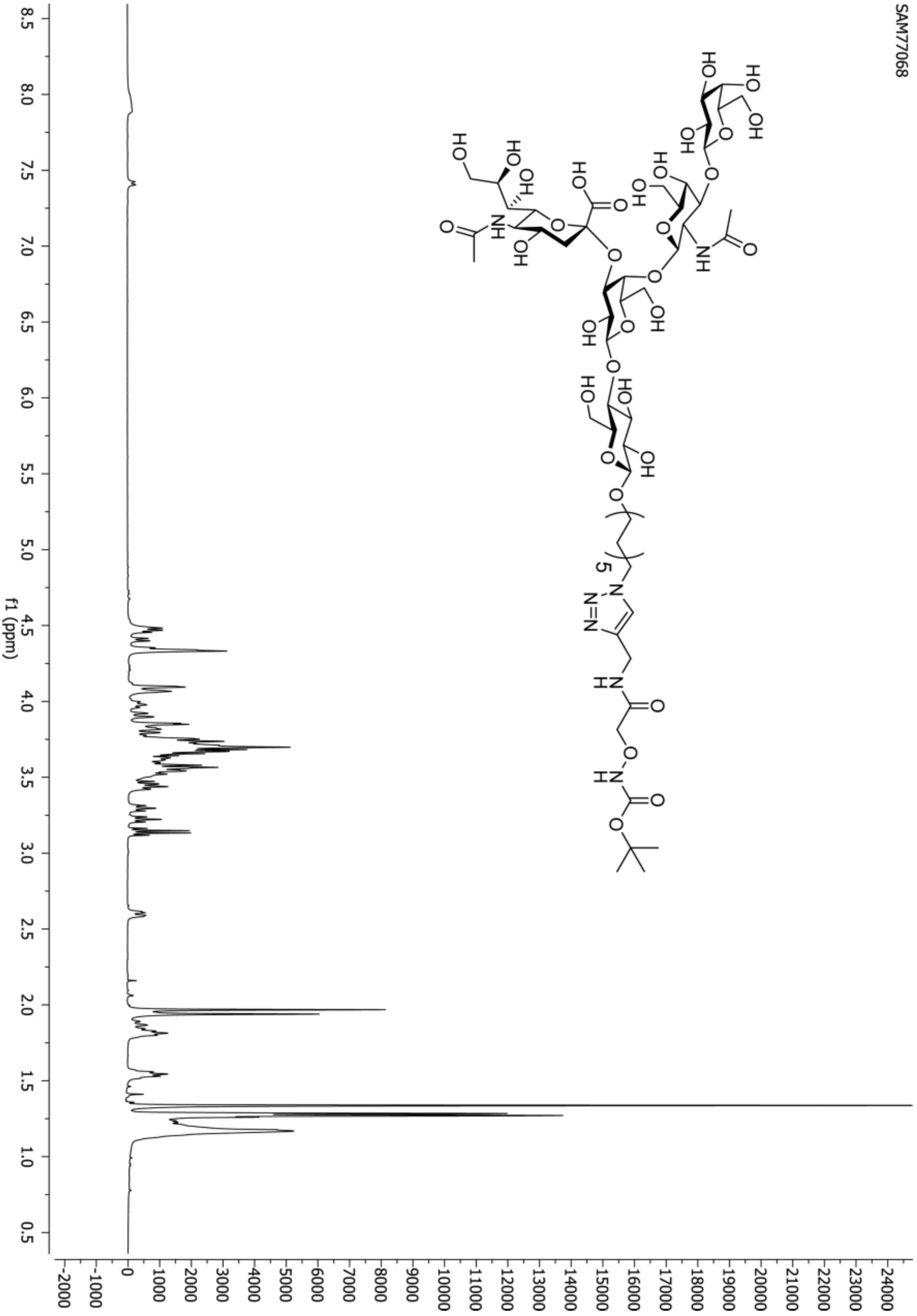

SAM77068
f1 (ppm)

DNA mutations, protein overexpression and purification

The gene for El Tor CTB was introduced into the multiple cloning site of a pMAL-p5X plasmid and the MBP gene was removed, to create plasmid pSAB2.2. Site-directed mutagenesis was performed to introduce a W88E mutation, which would remove the carbohydrate binding capacity of the protein, producing plasmid pTRB-W88E.

The W88E mutant protein was overexpressed in *E.coli C41* cells by induction with IPTG and growth at 30 °C for 24 h. After precipitation by ammonium sulfate (60%), the protein was redisolved in *phosphate buffer* (0.1 M sodium phosphate, 0.1 M NaCl, pH 7) and purified by nickel affinity chromatography (wash with 20 mM $NiSO_4$ and elute with 500 mM $NiSO_4$). SEC was used to isolate the mutant protein W88E (2.5 mL injected on to a Superdex™ 200 HiLoad™ 26/60 column, with the flow rate at 1.0 mL min^{-1}). ESI-MS (Bruker Daltonics MicroTOF) was used to confirm the mutations in the protein: W88E was found to have a mass of 11585.0 Da (calculated 11584.9 Da).

Protein sequences

The amino acid sequences are shown below for the CTB wild-type protein and the W88E mutant. The mutation from the wild-type sequence is underlined and highlighted in red:

	1	11	21	31
**CTB**	TPQNITDLCA	EYHNTQIYTL	NDKIFSYTES	LAGKREMAII
**W88E**	TPQNITDLCA	EYHNTQIYTL	NDKIFSYTES	LAGKREMAII

	41	51	61	71
**Wild-type**	TFKNGAIFQV	EVPGSQHIDS	QKKAIERMKD	TLRIAYLTEA
**W88E**	TFKNGAIFQV	EVPGSQHIDS	QKKAIERMKD	TLRIAYLTEA

	81	91	101
**wild-type**	KVEKLCVWNN	KTPHAIAAIS	MAN
**W88E**	KVEKLCVENN	KTPHAIAAIS	MAN

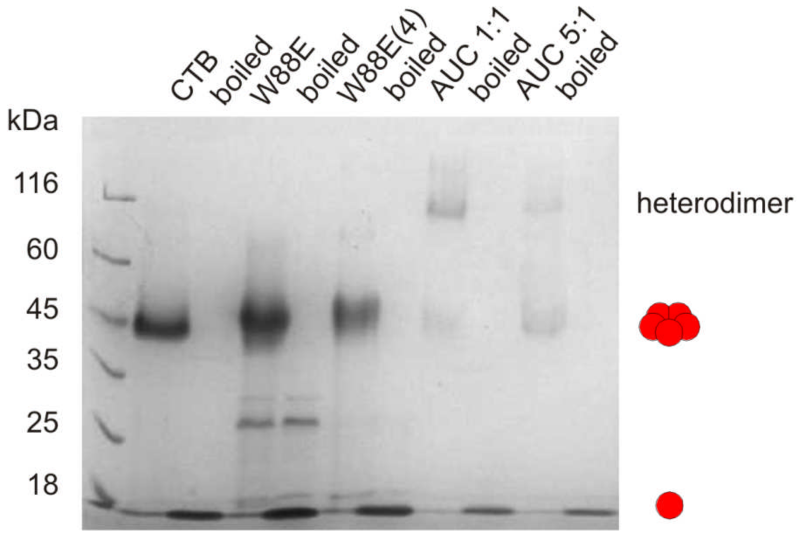

Figure S1 SDS-PAGE of wild-type CTB, mutant W88E and the pentavalent ligand **W88E(GM1)** (labelled as W88E(4)). The proteins migrate as stable protein pentamers on the gel, with a similar apparent mass of around 45 kDa. Samples labelled as "boiled" were heated to 95 °C and all were seen to denature into protein monomers. The samples from the AUC experiment show heterodimer formation in the 1:1 sample and some dimer in the 5:1 sample although due to the excess CTB there is a stronger pentamer band. The impurity in the W88E samples at ~25 kDa is FKBP-type peptidyl-prolyl cis-trans isomerase, which was not removed after the initial purification of W88E.

Protein modification procedure to make W88E(GM1)

All reactions were conducted in 0.1 M sodium phosphate, 0.1 M NaCl, pH 7. The N-terminus of the W88E protein was oxidised according to the procedure of Chen *et al*.[3] Methionine (15 µL, 200 mM, 10 equivalents) was added to CTB-W88E (500 µL, 600 µM), followed by the addition of $NaIO_4$ (30 µL, 50 mM, 5 equivalents). After 5 min at room temperature the reaction was complete and the oxidised protein was purified on a PD G-25 minitrap column to remove the $NaIO_4$, yielding oxidised W88E. **ESI-MS**: hemihydrate found $[M+11H]^{11+}$ 1051.60, $[M+10H]^{10+}$ 1156.68, $C_{508}H_{814}N_{138}O_{159}S_5$ requires $[M+11H]^{11+}$ 1051.71, $[M+10H]^{10+}$ 1156.79.

The Boc group was removed from **3** (1.0 mg, 0.7 µmol) by the addition of TFA (100 µL, 1.3 mmol) and stirring for 30 min before the TFA was evaporated under N_2 resulting in deprotected ligand **5**. As the unprotected aminooxy group is highly reactive, it was used immediately without further purification.

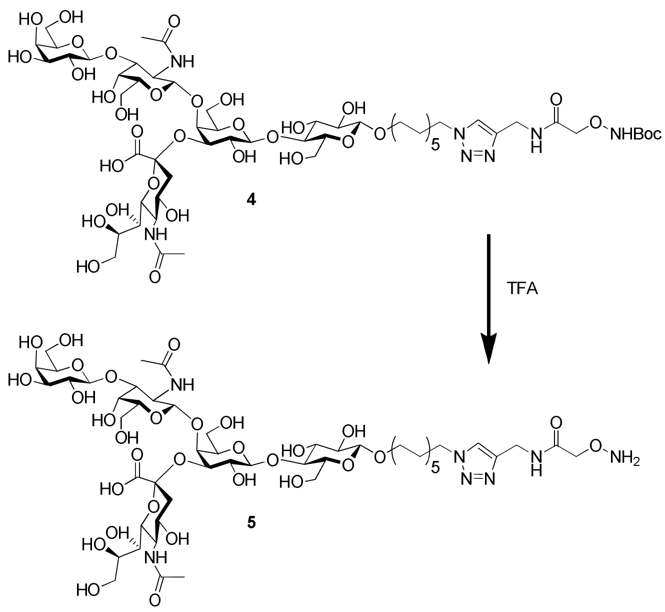

Oxidised W88E (500 µL, 600µM) and aniline (4.5 µL, to give a final concentration of 100 mM) were added to aminooxy-containing ligand **5** (10 equiv) and incubated at room temperature. The reaction was monitored by LC-MS until seen to be complete (20 h). The resulting neoglycoprotein was purified two times by SEC on a PD G-25 minitrap column to ensure complete removal of the unreacted oxyamine and aniline, yielding **W88E(GM1)** with a mass of 12844.5 Da (calculated 12844.4 Da) (Fig. S2). **ESI-MS**: found $[M+10H]^{10+}$ 1285.46, $[M+9H]^{9+}$ 1428.17, $C_{561}H_{901}N_{145}O_{188}S_5$ requires $[M+10H]^{10+}$ 1285.34, $[M+9H]^{9+}$ 1428.05.

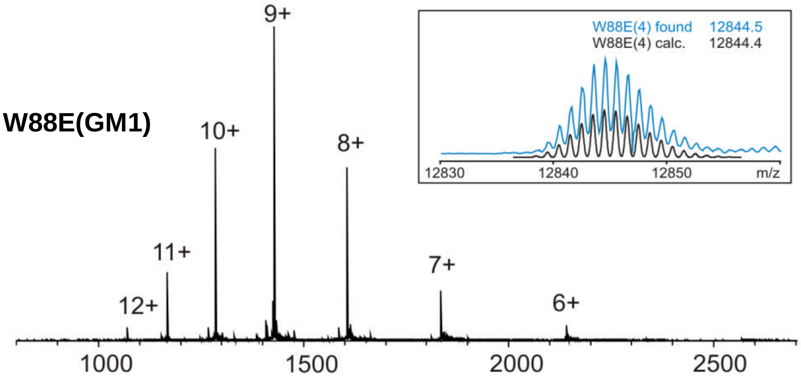

Figure S2 ESI-MS for **W88E(GM1)**, with inset showing the deconvoluted result.

Enzyme-linked Lectin assay (ELLA)

ELLA assays were performed using either ortho-phenylenediamine (OPD) or Amplex Red to detect the presence of CTB-conjugated horse radish peroxidase (CTB-HRP). All reagents were obtained from Sigma-Aldrich.

OPD Method

96-well polystyrene plates (NUNC Maxisorp) were prepared for the simultaneous testing of multiple samples. Ganglioside GM1 (100 µL, 1.3 µM in ethanol) was coated onto each well of a 96-well microtiter plate and the solvent was left to evaporate. The plate was washed with PBS (3 × 200 µL) to remove any unattached GM1 and any remaining free binding sites in the wells were blocked with BSA by incubating with a PBS solution containing 1% (w/v) BSA (100 µL) for 30 min at 37 °C. The wells were then washed again with PBS (3 × 200 µL). A series of inhibitor samples spanning a range of concentrations were prepared in PBS containing 0.1% BSA and 0.05% Tween 20. The samples were analysed in triplicate, initially over a large concentration range using a 10-fold dilution. A more accurate 2-fold dilution was then performed around the expected IC_{50} values. Each sample was mixed with CTB-HRP in the same buffer to give a final CTB-HRP concentration of 25 ng mL^{-1}. These mixtures of inhibitor and toxin were incubated at room temperature for 2 h before 200 µL was transferred to each GM1 coated well. The limits of detection were determined by control samples containing only the CTB-HRP component and no inhibitor, which gave the highest response, and a blank sample of only buffer which gave the lower limit. These two measurements were used to find the maximum and minimum optical density values for CTB-HRP binding to the GM1-coated wells. The inhibitor-toxin mixtures were incubated at room temperature for 30 min before the plate was washed with PBS, 0.1% BSA, 0.05% Tween 20 (3 × 200 µL), to remove the unbound CTB-HRP-inhibitor complexes. An ortho-phenylenediamine (OPD) solution was freshly prepared (25 mg OPD.2HCL, 7.5 mL 0.1 M citric acid, 7.5 mL 0.1 M sodium citrate and 6 µL of a 30% H_2O_2 solution, pH 6.0) and added to each well (100 µL). The solution was allowed to react in the dark, at room temperature, for 25 min before the oxidation reaction was quenched by the addition of 1 M H_2SO_4 (50 µL). The absorbance at 490 nm was measured within 5 min.

Amplex Red method

96-well high binding black flat-bottomed plates (Greiner 655077) were coated with ganglioside GM1 in the same way as described above. Inhibitor samples were prepared as above, except a 3-fold dilution series was set up across the full inhibitor concentration range in V-bottomed polypropylene 96-well plates (Greiner 651201) and the final CTB-HRP

concentration was 2.5 ng mL^{-1}. After incubating and washing the plates as above, the Amplex Red solution (100 µL per well; 5 µM Amplex Red, 5 µM H_2O_2 in phosphate-buffered saline) was added and fluorescence was monitored at 25 °C over 100 min in a Perkin-Elmer Envision plate reader (excitation 531 nm; emission 595 nm). Initial rates were used if the resulting plots deviated from linearity.

Data Processing

Samples were analysed in triplicate. Anomalous data points were removed in a few cases leaving duplicate results. The error of each sample was calculated as the standard error (Equation 1).

Equation 1

$$\text{standard error}=\frac{\sqrt{\frac{1}{N-1}\sum_{i=1}^{N}(x_i-\bar{x})^2}}{\sqrt{N}}$$

where N is the size of the sample, x_i is the observed value of each sample and $\bar{x}$ is the mean value of the sample.

The absorbance of each sample was converted to percentage binding by comparison with the maximum absorbance and minimum absorbance obtained from the control samples. These values were then plotted against log(concentration) of inhibitor for each sample and curve fitting was performed using the standard dose-response model in Origin (Equation). Fitting was performed with weighting on the data points from the standard error. Concentration values were converted to log(concentration) before curve fitting.

Equation 2

$$y = A1 + \frac{A2 - A1}{1 + 10^{(logx0-x)p}}$$

Where A_1 is y-value for the bottom plateau, A_2 is the y-value for the top plateau, x_0 is the IC_{50} value, x is log(inhibitor concentration) and p is the Hill slope parameter. IC_{50} values obtained from each method for **W88E(GM1)** agreed within 0.1 nM.

Isothermal titration calorimetry (ITC)

ITC titrations were performed using a Microcal ITC_{200} calorimeter (GE Healthcare) with a cell volume of 0.2028 mL and data was processed and fitted using Origin with the standard Wiseman one site model.[4] Protein samples were dialysed into *phosphate buffer* (0.1 M sodium phosphate, 0.1 M NaCl, pH 7) using SnakeSkin® pleated dialysis tubing (Thermo Scientific, 7000 MWCO) prior to analysis. Ligands were then dissolved in the dialysis buffer to ensure an exact match for the buffers during the titration. The reference cell was filled with water and the analysis cell was filled with the protein solution and both were allowed to reach thermal equilibrium at 25 °C before titrations were performed. Titrations typically comprised of 20 injections of 2 µL at 2 minute intervals. Separate titrations of the ligand into *phosphate buffer* were used to subtract the heat of dilution from the integrated data prior to curve fitting.

W88E(GM1) (43 µM) was titrated into a solution of CTB (4 µM) and an apparent K_d was detected of 30 nM. Monovalent GM1 azide **2** (50 µM) was titrated into CTB (5 µM) and had a K_d of 56 nM. The ITC titration of CTB and **W88E(GM1)** was also performed with the components in the cell and syringe reversed, i.e., CTB (50 µM) was injected into ligand **W88E(GM1)** (5 µM).

Table S1 ITC results for ligand **W88E(GM1)** binding to CTB compared to GM1os.

Cell component	Syringe component	K_d / nM	ΔH° / kcal mol^{-1}	n
CTB	**W88E(GM1)**	29.6 ± 7.5	-10.48 ± 0.98	1.11
**W88E(GM1)**	CTB	32.4 ± 10.7	-15.39 ± 1.71	0.54
CTB	GM1 azide **2**	55.6 ± 6.4	-14.92 ± 0.15	0.91

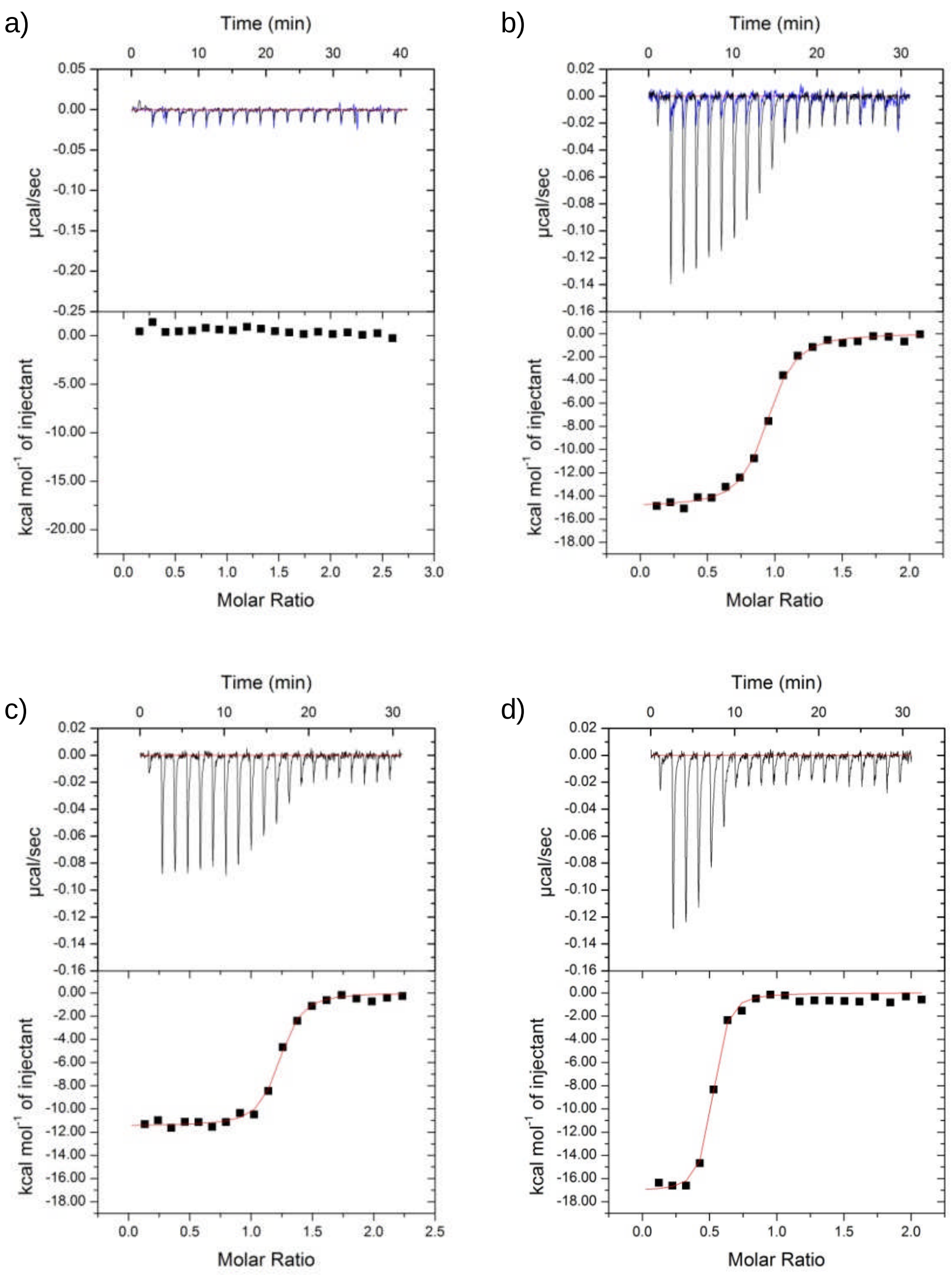

Figure S3 ITC plots for a) GM1os titrated into **W88E**; b) GM1 azide **2** titrated into CTB; c) **W88E(GM1)** titrated into CTB; d) CTB titrated into **W88E(GM1)**.

Analytical ultracentrifugation (AUC)

Protein samples in 0.1 M sodium phosphate, 0.1 M NaCl, pH 7 (1:1 20 µM each component, 5:1 20 µM CTB and 4 µM **W88E(GM1)**, 0.41 mL total volume) were centrifuged in 1.2 cm pathlength 2-sector aluminium centrepiece cells (sample in right hand sector, reference buffer in left hand sector) built with sapphire windows in an 8-place An50Ti or 6-place An60Ti analytical rotor running in an Optima XL-I analytical ultracentrifuge (Beckman Instruments, Inc., Palo Alto, California 94304) at 35 krpm and at a temperature of 20 °C. Changes in solute concentration were detected by 150 absorbance scans measured at 280 nm over a period of 10 hours.

Analysis and fitting of the data was performed using the software SedFit.[5] A continuous c(s) distribution model was fitted to the data, taking every 2nd scan. The resolution was set at 200 over a sedimentation coefficient range of 0.1-15.0 S. Parameters were set for the partial specific volume as 0.73654, the buffer density of 1.04910 and the buffer viscosity at 0.01410 for 0.1 M sodium phosphate, 0.1 M NaCl, pH 7. The frictional coefficient, the baseline and the raw data noise were floated in the fitting. The meniscus and bottom of the cell path were also floated after initial estimations from the raw data.

Dynamic light scattering (DLS)

Disposable polystyrene cuvettes were used for light scattering experiments performed using a Zetasizer Nano ZS (Malvern) at 20 °C measuring the scattering at an angle of 173° and using a laser wavelength of 633 nm. Protein samples (50-200 µL, 50-500 µM) in 0.1 M sodium phosphate, 0.1 M NaCl, pH 7, were filtered through a 0.8 µm filter (Millipore membrane filters) prior to measurements. Readings of 10 scans over 10 seconds each were taken in triplicate with the average calculated and measurement data was analysed using the Malvern volume percent function.

A peak at 8.4 nm was observed when **W88E(GM1)** and CTB were mixed at a ratio of 1:1. When the ratio of CTB: **W88E(GM1)** was increased to 5:1 the peak shifted to 7.8 nm suggesting that the data analysis could not differentiate between the two species of similar size in comparison to the AUC data. But no larger species were observed.

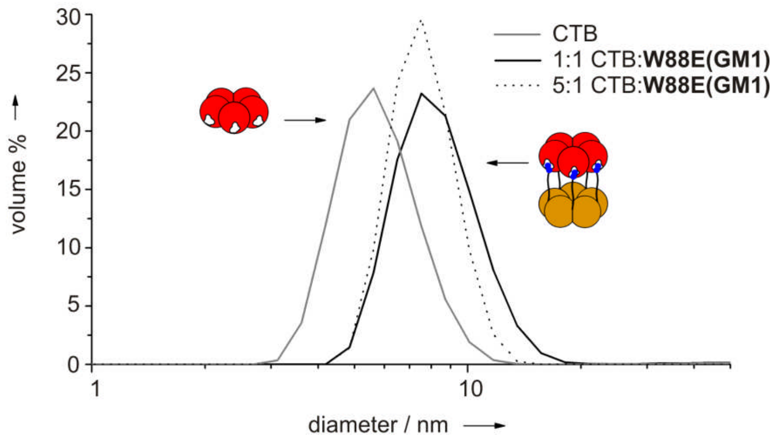

Figure S4 DLS plot of **W88E(GM1)** mixed with CTB.

References

[1] A. V. Pukin, C. A. G. M. Weijers, B. van Lagen, R. Wechselberger, B. Sun, M. Gilbert, M.-F. Karwaski, D. E. A. Florack, B. C. Jacobs, A. P. Tio-Gillen, A. van Belkum, H. P. Endtz, G. M. Visser, H. Zuilhof, *Carbohydr. Res.* **2008**, *343*, 636-650.

[2] E. Trévisiol, E. Defrancq, J. Lhomme, A. Laayoun, P. Cros, *Eur. J. Org. Chem.* **2000**, *2000*, 211-217.

[3] J. H. Chen, W. G. Zeng, R. Offord, K. Rose, *Bioconjugate Chem.* **2003**, *14*, 614-618.

[4] T. Wiseman, S. Williston, J. F. Brandts, L.-N. Lin, *Anal. Biochem.* **1989**, *179*, 131-137.

[5] P. Schuck, *Biophys. J.* **2000**, *78*, 1606-1619.
